# A Child with Psoriasis, Hypogammaglobulinemia, and Monosomy 7-Positive Myelodysplastic Syndrome

**DOI:** 10.4274/tjh.2014.0308

**Published:** 2015-02-15

**Authors:** Namık Özbek, Arzu Yazal Erdem, Özlem Arman Bilir, Fatma Karaca Kara, Mutlu Yüksek, Neşe Yaralı, Meltem Özgüner, Nazmiye Yüksek, Bahattin Tunç

**Affiliations:** 1 Ankara Children’s Hematology and Oncology Hospital, Clinic of Pediatric Hematology, Ankara, Turkey; 2 Ankara Children’s Hematology and Oncology Hospital, Clinic of Biochemistry, Ankara, Turkey; 3 Bülent Ecevit University Faculty of Medicine, Department of Children’s Immunology, Ankara, Turkey; 4 Yıldırım Beyazıt University Faculty of Medicine, Department of Histology Embryology, Ankara, Turkey; 5 Bülent Ecevit University Faculty of Medicine, Department of Pediatric Hematology, Ankara, Turkey

**Keywords:** Psoriasis, Hypogammaglobulinemia, monosomy 7, MDS

## TO THE EDITOR

A 3.5-year-old girl was admitted to our hospital with psoriasis, hypogammaglobulinemia, and pancytopenia present since 2 years of age. Before admission, due to the decreased number of B-lymphocytes and decreased immunoglobulin (Ig) levels (IgG: 196 mg/dL, IgM: 18.1, IgA: 26.8 mg/dL), she was diagnosed with autosomal recessive hypogammaglobulinemia and received intravenous Ig every month. She was referred to us after detection of 12% blasts and monosomy 7 in a bone marrow (BM) aspiration specimen. Her parents were cousins and 2 elder sisters had died of infection at 3 and 6 months of age, one of whom had had pancytopenia and hepatosplenomegaly. Physical examination revealed normal growth and diffuse psoriatic lesions. Laboratory investigations revealed hemoglobin of 8.5 g/dL, mean corpuscular volume of 92 fL, reticulocyte count of 0.4%, leukocyte count of 3.7x109/L (absolute neutrophil count of 0.4x109/L), and platelet count of 28x109/L with 2% blasts on peripheral smear. Lymphocyte subset analysis revealed 1% CD19+ cells. T-lymphocyte stimulation by phytohemagglutinin disclosed normal results. Virological, microbiological, and immunological studies; DEB test; and ferritin, vitamin B12, and folic acid levels were all unrevealing.

Bone marrow aspiration revealed 15% blasts and BM biopsy revealed 10%-15% CD34+ blastic cells and trilineage dysplasia. A clonal population with monosomy 7 was detected in 70% of metaphases studied. She was diagnosed with myelodysplastic syndrome (MDS) and refractory anemia with excess of blasts (RAEB). After 3 months, a BM study before BM transplantation (BMT) showed 23% blasts, revealing progression to acute myeloblastic leukemia (AML). After AML-BFM induction treatment, she underwent BMT from her fully matched uncle. Interestingly, her psoriatic lesions disappeared, and she is doing well 12 months after the BMT.

The only report in the literature similar to ours presented 3 children with diagnoses of hypo-/agammaglobulinemia and B-lymphocytopenia, followed by refractory anemia and monosomy 7 [1]. One of them progressed to RAEB after 6 years, similar to our patient progressing to AML. Another report described secondary transient MDS in a patient with X-linked agammaglobulinemia and a clonal abnormality in the BM [del(6q), -9 and der(11)] that disappeared within 1.5 years [2]. Secondary MDS or features mimicking MDS may be seen during viral infections, in patients with genetic abnormalities including microdeletion 22q11.2, and in nonmalignant disorders like juvenile rheumatoid arthritis, polyarteritis nodosa, and idiopathic thrombocytopenic purpura [3,4,5,6,7]. Informed consent was obtained.

A current hypothesis claims that in genetically predisposed persons, nonspecific stimulation of T-cells amplifies epidermal growth in psoriasis [8]. In the literature, the only relevant paper revealed MDS in an adult with psoriasis that occurred after etanercept treatment [9]. Although the number, distribution, and activity of T-cells were normal in our patient, the disappearance of psoriatic lesions after BMT may indicate an intrinsic T-cell defect,which is also a new finding. The promising outcome of BMT in our patient may also indicate the role of allogeneic mesenchymal stem cells in the healing of psoriasis. A recent study reported aberrant proliferative activity, increased apoptosis rate, and different gene expression profiles in bone marrow mesenchymal stem cells (BMMSCs) obtained from psoriatic patients, which lead to defective immune response [10]. The disappearance of psoriatic lesions in our patient may have been due to the immunomodulatory effect of allogeneic BMMSCs.

## References

[1] Srivannaboon K, Conley ME, Coustan-Smith E, Wang WC (2001). Hypogammaglobulinemia and reduced numbers of B-cells in children with myelodysplastic syndrome. J Pediatr Hematol Oncol.

[2] Narula G, Currimbhoy Z (2008). Transient myelodysplastic syndrome in X-linked agammaglobulinemia with a novel Btk mutation. Pediatr Blood Cancer.

[3] Yetgin S, Çetin M, Yenicesu İ, Özaltın F, Uçkan D (2000). Acute parvovirus B19 infection mimicking juvenile myelomonocytic leukemia. Eur J Hematol.

[4] Özbek N, Derbent M, Olcay L, Yılmaz Z, Tokel K (2004). Dysplastic changes in the peripheral blood of children with microdeletion 22q11. 2. Am J Hematol.

[5] Yetgin S, Ozen S, Saatci U, Bakkaloglu A, Besbas N, Kirel B (1997). Myelodysplastic features in juvenile rheumatoid arthritis. Am J Hematol.

[6] Yetgin S, Ozen S, Yenicesu İ, Çetin M, Bakkaloğlu A (2001). Myelodysplastic features in polyarteritis nodosa. Pediatr Hematol Oncol.

[7] Olcay L, Yetgin S, Okur H, Erekul S, Tuncer M (2000). Dysplastic changes in idiopathic thrombocytopenic purpura and the effect of corticosteroids to increase dysplasia and cause hyperdiploid macropolycytes. Am J Hematol.

[8] Bos JD, Rie MA, Teunissen MB, Piskin G (2005). Psoriasis: dysregulation of innate immunity. Br J Dermatol.

[9] Knudson RM, Tefferi A, Pittelkow MR, Davis MD (2011). Development of myelodysplastic syndrome evolving to acute myeloid leukemia in a patient receiving etanercept for psoriasis. J Am Acad Dermatol.

[10] Hou R, Liu R, Niu X, Chang W, Yan X, Wang C, Li J, An P, Li X, Yin G, Zhang K (2014). Biological characteristics and gene expression pattern of bone marrow mesenchymal stem cells in patients with psoriasis. Exp Dermatol.

